# This Better Be Interesting: A Speaker’s Decision to Speak Cues Listeners to Expect Informative Content

**DOI:** 10.1162/opmi_a_00058

**Published:** 2022-09-01

**Authors:** Hannah Rohde, Jet Hoek, Maayan Keshev, Michael Franke

**Affiliations:** Department of Linguistics & English Language, University of Edinburgh, Edinburgh, UK; Department of Language & Communication, Radboud University, Nijmegen, The Netherlands; Department of Linguistics, University of Massachusetts, Amherst, USA; Department of Linguistics, University of Tübingen, Tübingen, Germany

**Keywords:** language processing, pragmatics, predictability, informativity, real-world plausibility

## Abstract

In anticipating upcoming content, comprehenders are known to rely on real-world knowledge. This knowledge can be deployed directly in favor of upcoming content about *typical situations* (implying a transparent mapping between the world and what speakers say about the world). Such knowledge can also be used to estimate the likelihood of speech, whereby *atypical situations* are the ones newsworthy enough to merit reporting (i.e., a nontransparent mapping in which improbable situations yield likely utterances). We report four forced-choice studies (three preregistered) testing this distinction between situation knowledge and speech production likelihood. Comprehenders are shown to anticipate situation-atypical meanings more when guessing content (a) that a speaker announces (rather than thinks), (b) that is said out of the blue (rather than produced when prompted), and (c) that is addressed to a large audience (rather than a single listener). The findings contrast with prior work that emphasizes a comprehension bias in favor of typicality, and they highlight the need for comprehension models that incorporate expectations for informativity (as one of a set of inferred speaker goals) alongside expectations for content plausibility.

## INTRODUCTION

The process of producing natural language requires making a number of informational decisions, both about what content to express and how much detail to include. These decisions reflect well-studied pressures related to efficiency and expressivity (e.g., Degen et al., 2020; M. C. Frank & Goodman, 2012; Franke & Jäger, 2016; Grice, 1975; Levy & Jaeger, 2007; Rubio-Fernandez, 2016), which are captured in generalizations about cooperative speakers for whom what is not said is the obvious (Atlas & Levinson, 1981; Levinson, 2000). Content decisions have primarily been studied in contexts in which a speaker’s productions are already underway (e.g., modifier inclusion/omission and choices among semantically equivalent complex/simple predicates for M/I [manner/informativeness] implicatures) rather than content selection when a speaker is deciding whether to speak at all. If one way that an utterance can be relevant to the discourse is via its newsworthiness and if speakers therefore have a bias toward producing informative and newsworthy content, a concomitant comprehension bias ought to arise such that listeners come to expect newsworthy content.^1^

To illustrate, consider the passages about housing prices in example (1) and whether comprehenders have different expectations for a value that denotes what Sue *thinks* someone paid (something close to the average housing price?) versus what Sue believes would be newsworthy enough to merit *telling* (something more extreme than the average?).

(1)  a. Sue lives in New York. She **thinks** that her new neighbors bought their apartment for $___
    b. Sue lives in New York. She **told me** that her new neighbors bought their apartment for $___

If there is no distinction between what a speaker thinks and what they say out loud, then the completions for (1-a) and (1-b) ought to align. On the other hand, if comprehenders think that speakers in communicative contexts will use language to convey newsworthy content, then the context that emphasizes information exchange ([1-b] *She told me*) ought to elicit more extreme values than one without such emphasis ([1-a] *She thinks*). Note that (1-a) and (1-b) are both communicative contexts in that there is an author/narrator producing information about Sue in both cases. If comprehenders expect newsworthiness from language, then both (1-a) and (1-b) may induce a preference for a value that deviates from the average housing price, but the prediction is that such a preference ought to be stronger in the context that more explicitly emphasizes information exchange. Current models of language comprehension portray a close link between what comprehenders know about the world and the kinds of sentences they expect to encounter, insofar as sentences about situation-typical meanings are reported to be easier to process than situation-atypical meanings (e.g., Kutas & Hillyard, 1980). Such models do not deny a role for informativity or, more generally, relevance, but by emphasizing a comprehension preference for typicality and plausibility, they in effect depict language as a transparent modality that speakers use to convey what they observe in the world. In contrast, the approach we take here highlights the importance of speaker goals: In contexts where newsworthiness is a plausible speaker goal, models ought to make explicit a distinction between the prior probability of a certain meaning and the (inversely related) likelihood of a speaker choosing to produce an utterance to convey that meaning.

Modeling speaker goals—and comprehenders’ inferences about those goals—is fundamental to work on experimental pragmatics (A. Frank & Jaeger, 2008; M. C. Frank & Goodman, 2012; Sperber & Wilson, 1995). We follow researchers like A. Frank and Jaeger (2008) and M. C. Frank and Goodman (2012) in taking an information-theoretic approach to message encoding and decoding. Such an approach is apparent in a number of processing models, particularly those for speech production (Aylett & Turk, 2004; Gahl, 2008; Hale, 2006; Jurafsky et al., 1998; Levy & Jaeger, 2007; Piantadosi et al., 2011; Zerkle et al., 2017) but has received less attention for modeling comprehension (cf. Rohde et al., 2021; Sedivy, 2003). Regarding speaker goals of newsworthiness, there is evidence that in production, speakers are more likely to mention elements that are real-world atypical—for example, object color (yellow vs. blue bananas; Engelhardt et al., 2006; Engelhardt & Ferreira, 2014; Rubio-Fernandez, 2016; Sedivy, 2003), object material (ceramic vs. wool bowls; Mitchell et al., 2013), or the instrument used for an action (stab with a knife vs. ice pick; Brown & Dell, 1987; Grigoroglou & Papafragou, 2016; Lockridge & Brennan, 2002). Brown and Dell’s (1987) classic production study on content selection shows that while a particular object (a knife) may be the (presumed) preferred instrument for stabbing, the mention of that typical instrument is dispreferred. Rather, it is only when a story involves an atypical stabbing (with an icepick) that speakers prefer to mention the instrument. If it is the case that listeners track these real-world priors and speech production likelihoods, then these probabilities should be reflected in their comprehension biases—we do not expect a speaker to have encountered an icepick stabbing (one hopes) or a blue banana or a woolen bowl, but we would expect them to mention it if they did.

The relationship between speakers’ productions and listeners’ interpretations in such contexts is well captured by models that are built on principles of rational communication (maxims of cooperative conversation (Grice, 1975) and later developments of generalized conversational implicatures (Levinson, 2000), the Rational Speech Act model (M. C. Frank & Goodman, 2012), rational redundancy (Degen et al., 2020), efficiency and pertinence (Rubio-Fernandez, 2016), and game theory (Benz et al., 2006; Franke, 2009). Such models are relevant to understanding speakers’ choice among available forms, as well as comprehenders’ response when such forms are used: see work on scalar implicatures (Augurzky et al., 2019; Hunt et al., 2013; Spychalska et al., 2016), particularly using EEG to test the interplay of prior and likelihood for scalars (Werning & Cosentino, 2017; Werning et al., 2019), and on M-implicatures (Bergen et al., 2016). However, few models explicitly include the speaker’s choice to speak up in the first place (but see Lassiter & Goodman, 2017; Rohde et al., 2021) and their prediction has not been tested empirically. However, these models usually consider cases where the speaker must choose a form to convey a given message, but not the decision of whether to speak or what message to convey in the first place, but see Rohde et al. (2021) for a recent account of explicit message choice framed within a Bayesian approach to informativity. In that approach, comprehenders’ processing of a particular form is influenced by two factors. One is the prior, the probability of a particular meaning, whereby more typical situations will have a higher prior. The other is the likelihood, the conditional probability of a speaker articulating a meaning given that that meaning holds; if one of the speaker’s goals is to be informative, atypical situations will have a higher likelihood of being mentioned.

There are several key insights afforded by this Bayesian conceptualization. First is that the prior and likelihood can each be considered in their own right—when a comprehender estimates the probability of encountering different utterances, their assessment reflects not only an estimate of whether the meaning is probable but also their estimate of whether a speaker would have selected a particular surface form to convey that meaning. Second is that the available surface forms can include silence. Indeed a comprehender should be surprised (and seek out alternative intended meanings) if a speaker formulates an utterance about content that is too easily inferable (see Kravtchenko & Demberg, 2015). Lastly, estimates of the prior and likelihood can be adjusted independently. The prior may shift if the context moves from the familiar real world to an alternative reality (e.g., Troyer & Kutas, 2018); the likelihood may adjust in more subtle ways depending on factors like who the speaker is, why they are speaking, or who they are speaking to. The studies presented here test this approach and contrast its predictions with those of a simpler model that only emphasizes typicality, with no difference predicted between comprehenders’ estimates of speakers’ thoughts and their utterances, as is implicit in comprehension models that link situation typicality directly to processing ease (Bicknell et al., 2010; Hagoort et al., 2004; Kuperberg, 2021; Kutas & Hillyard, 1980; Matsuki et al., 2011; Stanovich & West, 1979).

Prior work shows that comprehenders can favor messages that are sufficiently newsworthy to merit sending (faster reading times for a newsworthy message about socks that cost $100 than socks that cost $2; Rohde et al., 2021). While Rohde et al.’s reading-time results establish slower processing for situation-typical meanings compared with situation-atypical meanings, their studies do not probe the *content* of participants’ expectations—which meanings do comprehenders believe speakers are likely to have *encountered* in the world (the prior) versus have chosen to *talk* about (the likelihood) and what factors affect these expectations?

The studies presented here use forced-choice tasks to test comprehenders’ guesses about an upcoming numeric value in a proposition across conditions that vary the emphasis on information exchange. Experiment 1 manipulates the status of the proposition as either an individual’s internal thought versus an articulated utterance. Experiments 2 and 3 manipulate the context of production—a statement produced when prompted versus out of the blue and when addressed to a single listener versus a crowd. Experiment 4 combines the conditions in a single study, testing three conditions that vary the emphasis on information exchange. The results suggest that comprehenders estimate the likelihood of utterance production in favor of content that deviates from real-world priors and they do so in context-sensitive ways.

## EXPERIMENT 1: PRIOR VERSUS LIKELIHOOD

This first experiment tests comprehenders’ expectations about upcoming content when it constitutes a character’s reported thought versus their reported speech, see example (2).

(2)  Liam is a man from the US. Liam lives down the street from Rebecca.
    a. Rebecca **thinks** that Liam has … T-shirts.
    b. Rebecca **announced to me** that Liam has … T-shirts.
           **O** 21       **O** 29

We manipulate whether a character is said to think or announce something. Participants chose between a “low” value approximating the mean and a “high” one that is expected to be more newsworthy. If participants expect speakers to transparently map thoughts into speech, then a character’s reported thoughts ought to parallel that character’s reported speech. If, however, participants distinguish between the prior probability of a situation occurring and the likelihood that a speaker would choose to produce a sentence about that situation, the think condition ought to yield estimates that are closer to participants’ real-world priors than the announce condition.

Note that the paradigm we are using involves a character’s reported thoughts and speech, with an implicit narrator who is reporting these situations as in (2). It is also possible that participants will expect the narrator themselves to have something newsworthy to say, inducing expectations that both Rebecca’s thoughts and her announcements ought to be newsworthy. As we will show, despite this double-nesting, participants do distinguish the two conditions and favor the less real-world-typical value when the passage involves reported speech.

### Method

#### Materials.

Each of 12 experimental passages introduced an individual (Liam in (2)) and someone who would know that individual reasonably well (neighbor, Rebecca). The final sentence described this second person’s thought or announcement about some aspect of the first individual’s life (Appendix A in the Supplemental Materials). The manipulation here and in Experiments 2 and 3 was implemented as a within-participants and within-items design. The two numeric values for each passage were selected via a pretest (Appendix B in the Supplemental Materials) where participants provided free responses to questions about the number of items or frequency of events in someone’s life (*Liam is a man from the US. How many T-shirts does he have?*).

The “low” value was selected as a value slightly above that item’s pretest mean (mean + 1/5*standard deviation) and the “high” one as a value farther above the mean (mean + 4/5*standard deviation, with rounding strategy explained in Appendix B in the Supplemental Materials; see also Cummins, 2015).^2^

Both values were “plausible” in that they represented values in the range elicited in the pretest, but the high values were less probable (and therefore more newsworthy). Participants also saw eight filler passages: Four required speculation; four were catch trials with a correct answer (Appendix C in the Supplemental Materials). Participants who made mistakes on catch trials were excluded from analysis.

#### Participants.

Ninety-seven native-English speakers were recruited through Amazon Mechanical Turk and paid for their participation ($2). We excluded participants with catch trial mistakes, leaving 90 participants (mean age 41.1, range 23–77).

#### Data analysis.

For all experiments, we analyzed the binary outcome of participants’ forced-choice selection (low versus high value) with generalized logistic mixed effects models (GLMM: Jaeger, 2008) using the lme4 package (Bates et al., 2015) in R (R Core Team, 2019) with random slopes and intercepts of condition for participants and items (Barr et al., 2013). The significance of the categorical fixed effect of *condition* was determined via a likelihood ratio test comparing the fit of the model to one with the same random effects structure but no fixed effect.

### Results

**Figure 1.**
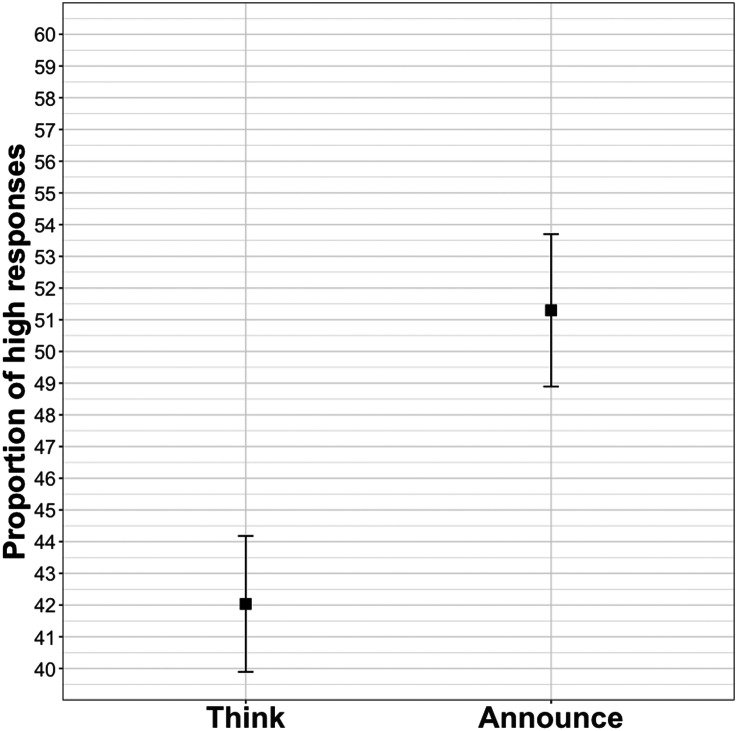
**Proportion of high responses in Experiment 1.** Error bars here and in other figures represent standard error of participant means.

The announce condition yielded more selections of the higher value than the think condition (*β* = 0.40, *SE* = 0.15, *z* = 2.66, *p* < .001). Figure 1 shows a preference for the lower, more typical, value in the think condition and a 50–50 split between the lower and higher values in the announce condition. (All materials, datasets, and analysis scripts for this and the following experiments can be found at https://osf.io/9eg34/.)

### Discussion

As predicted by a model in which expectations for newsworthiness influence comprehenders’ guesses about upcoming content, comprehenders showed a stronger preference for the situation-typical value (close to the estimated real-world mean) when the passage reported someone’s thoughts rather than their speech. The finding that the think condition showed a substantial rate of higher value responses could reflect participants’ low sensitivity to the contrast between the chosen numbers or their consideration that the think sentences were themselves utterance productions from a narrator and thus may contain information that is interesting enough to utter.

## EXPERIMENT 2: LIKELIHOOD OF SPEECH

If comprehenders estimate utterance likelihood when making guesses about upcoming content, a question is whether that likelihood is malleable. If it is, certain discourse contexts may increase the expectation for newsworthiness—for example, spontaneous speech would be predicted to contain more newsworthy content than speech that is produced as an answer to a question.^3^

### Method

#### Materials.

Thirty-five experimental passages followed the structure from Experiment 1, except that the final sentence varied whether the narrator reports that a character said something out of the blue or when asked (Appendix D in the Supplemental Materials).

(3)  Liam is a man from the US. Liam lives down the street from Rebecca. Last week,
    a. **when asked about it**, Rebecca said that Liam has … T-shirts.
    b. Rebecca **out of the blue** said that Liam has … T-shirts.
            **O** 21     **O** 31

As in Experiment 1, the values were selected via a free-prompt pretest (Appendix F in the Supplemental Materials). Here, the lower value corresponds to the mean of the pretest responses and the higher value to (approximately) the mean plus one *SD* of the pretest responses. The fillers matched those from Experiment 1.

#### Participants.

One hundred ten native speakers of English were recruited through Amazon Mechanical Turk and paid for their participation ($5). We excluded participants with catch trial mistakes, leaving 103 participants (mean age 37.7, range 19–68).

### Results

**Figure 2.**
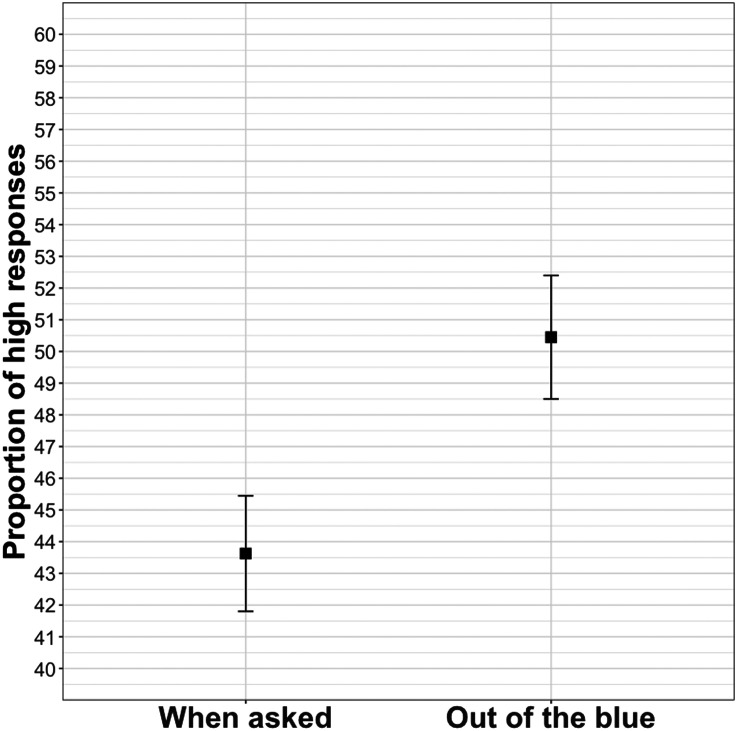
Mean proportion of high responses in Experiment 2.

As predicted, the out of the blue condition yielded more selections of the higher value than the when asked condition (*β* = −0.34, *SE* = 0.11, *z* = −3.16, *p* < .01; deviation coding was used for *condition* here and in Experiments 2 and 3). Figure 2 shows a preference for the lower, more typical, value in the when asked condition and a 50–50 split between the lower and higher values in the out of the blue condition.

### Discussion

Experiment 2 shows that comprehenders prefer the atypical (newsworthy) value more when a narrator reports on speech that is spontaneous. This finding is again in line with the informativity-driven model. While participants’ baseline prior is unlikely to be affected by our manipulations, our results show that the discourse context informs participants’ estimate of a speaker’s sentence, presumably via the likelihood. The fact that the when asked condition showed a substantial rate of higher value responses could, in addition to the reasons mentioned in Experiment 1, reflect participants’ guess that the posed question (*when asked*) itself presupposed some potential newsworthiness of the value.

The mean of the when asked condition aligns with that of the think condition in Experiment 1. This suggests that participants believe that answers to questions reflect what speakers think, which is in turn different from that they choose to talk about.

## EXPERIMENT 3: AUDIENCE SIZE

The third experiment tests whether comprehenders use information about the speaker’s audience to adjust their expectations about upcoming content. The larger the audience that a narrator describes, the more newsworthy the expected content of reported speech ought to be.^4^

### Method

#### Materials.

Thirty-five experimental passages were adapted from Experiment 2 such that the reported speech was said to me or to everyone (Appendix E in the Supplemental Materials).

(4)  Liam is a man from the US. Liam lives down the street from Rebecca. Last week at the conference,
    a. Rebecca said **to me** that Liam has … T-shirts.
    b. Rebecca stood up and said **to everyone** that Liam has … T-shirts.
          **O** 21      **O** 31

The numeric values were the same as in Experiment 2, as were the filler items.

#### Participants.

Two hundred three native speakers of English were recruited through Amazon Mechanical Turk and paid for their participation ($5). We excluded participants with catch trial mistakes, leaving 152 participants (mean age 37.2, range 22–71).

### Results

**Figure 3.**
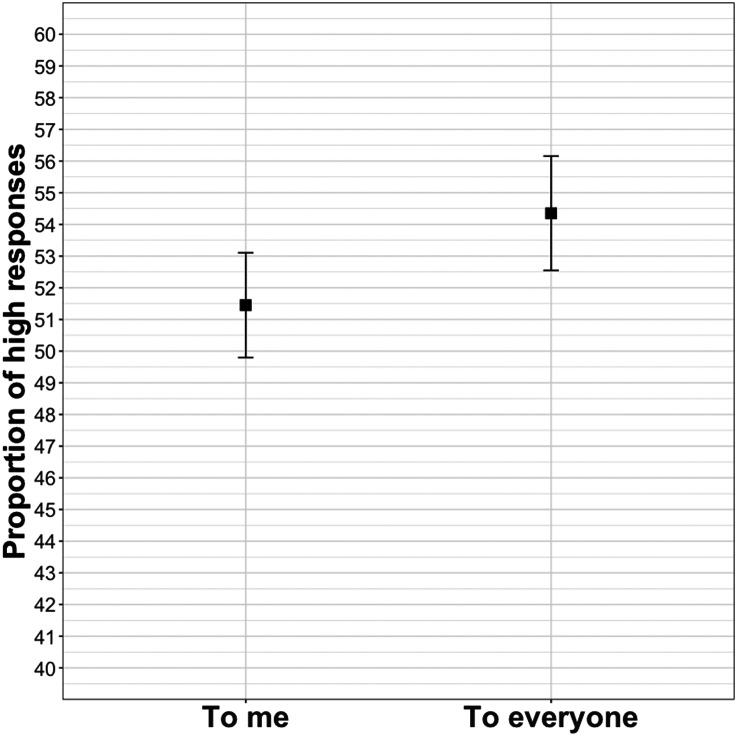
Mean proportion of high responses in Experiment 3.

As predicted, participants selected the higher value more in the to everyone condition than in the to me condition (*β* = 0.17, *SE* = 0.06, *z* = 2.59, *p* < .05). As can be seen in Figure 3, the effect, though statistically significant, is modest.

### Discussion

The results from Experiment 3 show that comprehenders expect the content of an utterance to be more newsworthy when a narrator describes that the content is shared with a large group of people rather than an audience consisting of a single person. This is in line with recent findings showing that manipulating the relationship between a speaker and addressee (stranger vs. family member) can alter comprehenders’ lexical predictions (Rubio-Fernandez et al., 2019). Comparing Figure 3 to Figures 1 and 2 shows that the proportion of high responses in the to me condition matches that of the announce condition from Experiment 1 and the out of the blue condition from Experiment 2. This is to be expected, since the prompts, though formulated slightly differently, correspond to similar conversational scenarios: a speaker, of their own volition, decides to convey a piece of information in an utterance to a (presumably) single other person.

## EXPERIMENT 4: VARIATION ACROSS THREE CONTEXTS

This experiment combines the conditions from Experiments 1–3 to create three levels of emphasis on information exchange. We vary the phrasing in order to avoid task-specific strategies that may have arisen in Experiments 1–3 from the lack of variation (in conditions and phrasing).^5^

### Method

#### Materials.

Forty-two experimental passages included 21 adapted from Experiments 2 and 3, plus 21 additional passages (Appendix G in the Supplemental Materials). Three conditions were devised based on the earlier studies’ manipulations.

(5)  Liam is a man from the US. Liam lives down the street from Rebecca.
    a. LOW: Last week, **when asked about it**, Rebecca said that Liam has … T-shirts.
    b. MID: Last week, Rebecca **announced** that Liam has … T-shirts.
    c. HIGH: Last week at the conference, Rebecca **stood up and said to everyone** that Liam has … T-shirts.
           **O** 18     **O** 28

The numeric values were derived via a free-prompt pretest (Appendix H in the Supplemental Materials). The lower value corresponds to the mean of the pretest responses and the higher value to (approximately) the mean plus one *SD* of the pretest responses. Each condition used two formulations, distributed between-items (LOW: *thought/when asked about it said*, MID: *announced/out of the blue said to me*, HIGH: *stood up and said to everyone/stood up and announced to the crowd*). Ten new fillers were added as attention checks (Appendix I in the Supplemental Materials).

#### Participants.

Three hundred native speakers of English were recruited through Prolific and paid for their participation (prorated at £7.50). We excluded participants with more than two attention check errors, leaving 275 participants.

### Results

**Figure 4.**
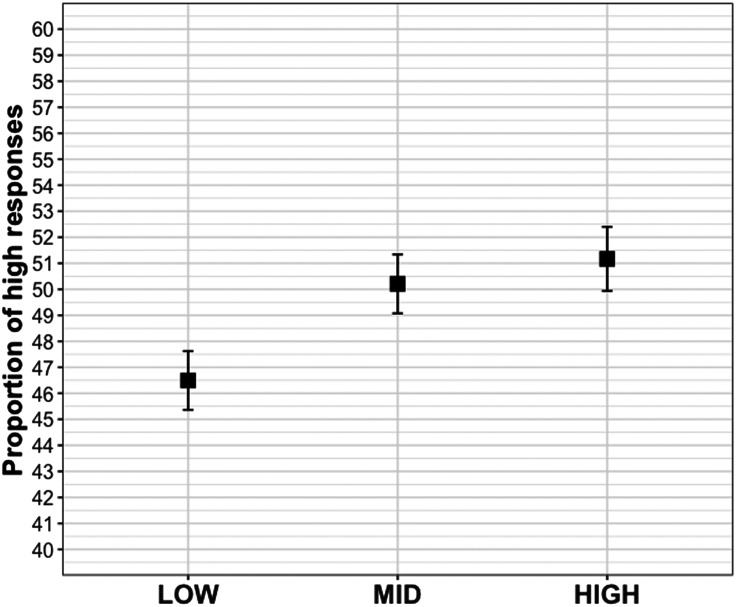
Mean proportion of high responses in Experiment 4.

Participants selected the higher value at different rates across conditions (*p* < .01; *condition* with baseline MID), with a significant difference between MID∼LOW (*β* = −0.17, *SE* = 0.06, *z* = −2.62, *p* < .01) but not MID∼HIGH (*β* = 0.05, *SE* = 0.07, *z* = 0.69, *p* = .49). See Figure 4.

### Discussion

Experiment 4 confirms that comprehenders’ expectations for newsworthy content is malleable, and it does so using a design that combines conditions from the previous three experiments. Specifically, the results show more high-value selections for the MID condition than the LOW condition: The lower informativity expression *thought* from Experiment 1 and *when asked* from Experiment 2 induce fewer selections of an atypical value. The MID condition contained expressions with some elements that emphasized information exchange (*announced* from Experiment 1 and *out of the blue* from Experiment 2) as well as one that deemphasized information exchange (*said to me*, as opposed to *said to everyone* from the HIGH condition). The LOW∼MID difference confirms that participants expect more newsworthy content when a speaker chooses to speak, rather than when they are thinking or being asked. The lack of MID∼HIGH difference may indicate that audience size has less of an impact, but it may also simply show that *speaking out of the blue* and *announcing* are cues to informativity that rival *speaking to a crowd*.

## GENERAL DISCUSSION AND CONCLUSION

Across four experiments, we measured comprehenders’ informativity expectations. Comprehenders favored an atypical (high) value more in passages that depict a speaker announcing something out loud (rather than thinking it), speaking out of the blue (rather than when asked), and, less consistently, when the speaker is depicted as addressing a large audience (rather than a single listener). The act of choosing to convey content in speech, as well as the context of that speech, affects comprehenders’ expectations. These findings can be captured in a Bayesian approach in which the probability comprehenders assign to a particular utterance rationally combines the probability of the described situation [*p*(*meaning*)] and the conditional probability that a speaker would articulate a linguistic form to describe such a situation to a certain audience [*p*(*form*|*meaning*)]. Our findings suggest that the prior and likelihood are separable and that the likelihood can be manipulated independently of the prior.

It is worth noting that although the observed effects are statistically robust, the numeric differences seem fairly small. Overall selection rates in this study were close to chance level (ranging between 42–55%). The relatively small difference between conditions could be related to the fact that the two values that participants had to choose between were relatively similar. Only one standard deviation distinguishes the typical and atypical values. Thus, it could be that participants are not fully aware of the contrast. It could even be that for some participants, the higher value is perceived as more probable, given that the higher values were provided by some participants in the pretests as their “best guess.” It is possible that with more prominently discriminated values, participants’ preferences would be even clearer. Another possibility is that participants perceived the low-informativity conditions (think, when asked, and to me) as still intended to be informative. Under a general presumption of relevance, participants would consider that there is a narrator, the experimenter, who reports the newsworthy thoughts and statements of different characters. A narrator could be relevantly informative by describing a character who thinks surprising thoughts or who boldly produces a highly uninformative utterance. Indeed, across experiments, the pretest participants produced values either below the lower response value or up to the halfway point between the lower and higher response values roughly 3/4 of the time (i.e., they favored “typical” values in the pretest task that did not emphasize information exchange), whereas the main-task participants chose the lower value closer to half the time. This may indicate that that the main task yielded a decreased preference for the typical values, possibly because all main-task conditions were “communicative” to some degree.

The contrast between the conditions in Experiment 3 was even smaller than in Experiments 1–2 and it did not replicate in Experiment 4. This could mean that the choice to spontaneously produce an utterance (rather than remaining silent) has more influence on informativity expectations than audience design considerations. However, it is also possible that the cues used in the Experiment 3 (and the MID and HIGH conditions in Experiment 4) all emphasize information exchange to some degree—either by invoking a narrator who themselves may be conveying information to the reader (“said to **me**”) or by describing bolder communicative acts (“stood up and said to everyone”), which perhaps are more likely to be retold by a narrator.

To address these issues, future studies should consider more direct assessment of listeners’ expectations of speaker content, ideally using first person speech (“I think Liam has … T-shirts”) and manipulating the speech scenarios in more direct ways that avoid the need for a narrator’s description of the situation. The goal would be to avoid the nested descriptions (“Rebecca thinks that Liam has … T-shirts”) and instead present participants with the communicative scenarios via videos or perhaps the use of confederates who produce the target sentences. As is, we cannot rule out an account in which participants are tracking the co-occurrence statistics of expressions like those in our materials rather than modeling the deeper reasoning behind speakers’ language production decisions. Our materials may have also introduced additional processing complexity via the double-nesting, which future work would be wise to avoid.

That said, our results are in line with a bias for newsworthiness (atypicality) in speaking. However, one might ask whether an expectation for accuracy (typicality) when thinking or answering could also explain our results. However, it is not clear why participants would not also expect accuracy when a speaker goes on record. Expectations for newsworthiness should not undermine expectations for accuracy; atypical meanings simply constitute content that is rare (but true) and whose rarity makes a speaker more likely to mention it.

To conclude, we argue that comprehenders consider both content plausibility and utterance likelihood, such that a “good” utterance is one that balances the prior probability of the content with its novelty. Our focus on content selection goes beyond prior studies of rational speaker-listener behavior, by considering message-level production choices rather than the inclusion/omission of linguistic elements, or the choice between semantically equivalent forms, once an utterance is already underway. In addition, we find context-driven effects on comprehenders’ estimates of utterance likelihood. The current study thus emphasizes the importance of including a bias for informativity in models of language comprehension, a bias that may pull linguistic expectations away from situation-typical content. Importantly, this bias is not a uniform one but varies systematically with the speaker’s context of use. This sets the stage for additional psycholinguistic research to consider different metrics of what makes language use efficient and relevant.

## ACKNOWLEDGMENTS

For the purpose of open access, the authors have applied a Creative Commons Attribution (CC BY) license to any Author Accepted Manuscript version arising from this submission.

## FUNDING INFORMATION

This work was financially supported by a grant awarded to MF by the German Research Council via the Priority Program Xprag.de (https://xprag.de/; DFG SPP 1727, FR 3482/1-2) and HR, Leverhulme Trust (https://dx.doi.org/10.13039/501100000275), Award ID: PLP-2016-128.

## AUTHOR CONTRIBUTIONS

HR: Conceptualization; Methodology; Writing - original draft; review & editing. JH: Conceptualization; Formal analysis; Methodology; Visualization; Writing - original draft; review & editing. MK: Conceptualization; Formal analysis; Methodology; Writing - review & editing. MF: Conceptualization; Methodology; Writing - review & editing.

## Notes

^1^ Language users of course do many things with language aside from conveying newsworthy information, but the use of language as a channel for relevant information transfer nonetheless represents a fundamental reason to communicate.^2^ It is worth highlighting that this simple operationalization in terms of empirical means and standard deviations may be problematic in the sense that these summary statistics are not meaningful in the same way for different kinds of distributions (see Appendix Figures 5, 6, and 7 in the Supplemental Materials).^3^ This experiment was preregistered: osf.io/dhm5g.^4^ This experiment was preregistered: osf.io/6t5ze.^5^ This experiment was preregistered: osf.io/xsjqn.

## Supplemental Materials

https://osf.io/9eg34/
