# Supplementary material for: This Better Be Interesting: A Speaker’s Decision to Speak Cues Listeners to Expect Informative Content
Source: Open Mind (Camb). 2022 Sep 1;6:118–31. doi: 10.1162/opmi_a_00058 (PMC9692056; doi:10.1162/opmi_a_00058)
Supplement: Supplementary file 1 [file opmi-06-118-s001.pdf]

Appendix A: Items experiment 1

burger Joseph is a man from the US. Joseph lives next door to Sue. Sue thinks / announced to me that Joseph ate . . . burgers last month. 8 / 11

calls Lisa is a woman from the US. Lisa has a friend, Kevin. Kevin thinks / announced to me that Lisa made . . . phone calls last week. 22 / 32

class Erin is a first grade student in primary school. Erin has an uncle, Josh. Josh thinks / announced to me that there are . . . children in Erin's class. 24 / 27

coffee Andy is a man from the US. Andy has an aunt, Katherine. Katherine thinks / announced to me that Andy drank . . . cups of coffee last week. 14 / 20

cook Tony is a man from the US. Nick has a sister, Emily. Emily thinks / announced to me that Tony cooked . . . meals at home last month. 12 / 17

facebook Judith is a woman from the US. Judith has a brother, Bill. Bill thinks / announced to me that Judith has . . . Facebook friends. 207 / 268

friends Lelia is a woman from the US. Lelia lives around the corner from Brandon. Brandon thinks / announced to me that Lelia has . . . friends. 10 / 14

hair Betty is a woman from the US. Betty works at an office with David. David thinks / announced to me that Betty washed her hair . . . times last month. 21 / 27

movie Nick is a man from the US. Nick went to school with Stephanie. Stephanie thinks / announced to me that Nick saw . . . movies last year. 22 / 36

restaurants Sarah is a woman from the US. Sarah has an acquaintance, Eric. Eric thinks / announced to me that Sarah went to . . . restaurants last year. 46 / 78

shoes Melanie is a woman from the US. Melanie has a colleague, Bob. Bob thinks / announced to me that Melanie owns . . . pairs of shoes. 73 / 152

tshirts Liam is a man from the US. Liam lives down the street from Rebecca. Rebecca thinks / announced to me that Liam has . . . T-shirts. 21 / 29

Appendix B: Experiment 1 pre-test

The pre-test for Experiment 1 was used to estimate participants' priors. The pre-test participants (N=31 after elimination of participants who failed the catch trials) did not participate in any other experiment in this paper. They were recruited on Amazon Mechanical Turk and paid $2. Each participant saw the same 12 target scenarios from the Experiment 1 materials. They were asked in a free-response task to answer questions about things like the number of objects in someone's possession or the frequency of events in someone's life (*Sarah is a woman from the US. How many restaurants did Sarah go to last year?*). When choosing the alternatives for experimental materials rounded values to the nearest whole number, except if one of the values for a specific item was a multiple of 5. In that case, we rounded one of the values to the closest other whole number to make sure that both values were either a multiple of 5 or not to avoid a confound where a precise (non-multiple-of-5) value seemed more informative. This reflects findings that 'random-seeming' numbers receive a more precise interpretation than numbers that are a multiple of 5 or 10 (e.g., Cummins 2015). Making sure that both values seem equally 'precise' avoids a potential confound of precise values (versus estimates) seeming more informative (for instance because saying that someone did 17 loads of laundry not only provides information about the number of times someone did laundry, but also implies that the speaker somehow counted/obtained/remembered the exact number). The distribution of the 31 responses for each item is shown in Figure 1.

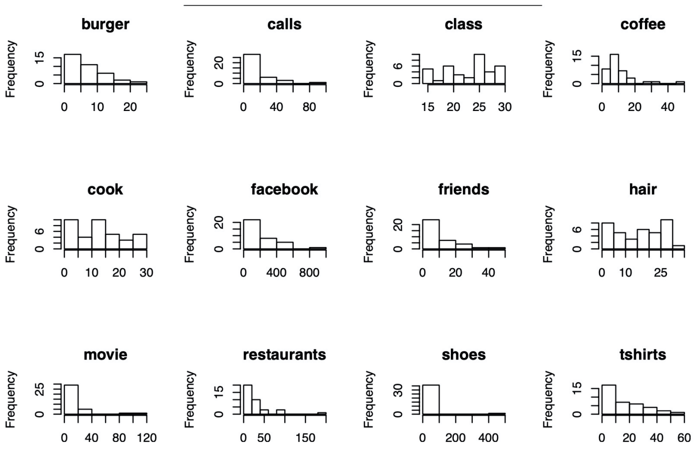

Figure 1: Frequencies of different values in pre-test norming for Experiment 1

Appendix C: Filler items (Experiments 1-3)

correct1 No one knows exactly when the Roman alphabet was first invented. The letters correspond roughly to spoken sounds but not exactly. There are ... letters in the modern alphabet. 26 / 70

correct2 Johnny and his brother are looking forward to the holidays. They've made a gingerbread house and decorated the tree. Johnny's favorite Christmas carol is 'The ... days of Christmas' 14 / 12

correct3 My best friend always reminds me to take a break. She quotes Roald Dahl that 'a little nonsense now and then, is cherished by the wisest men.' It's true that there are only ... hours in a day. 24 / 8

correct4 Everyone eventually has to leave home and make their way in the world. You have to face earning a living and doing your own laundry. You have to stand on your own ... feet. 6 / 2

speculation1 Corey and Charlotte are professional tuba players. They recently had a conversation about how old the tuba actually is. They concluded that the tuba is at most ... years old. 151 / 217

speculation2 The Grongitts went to a barbecue party again last weekend. Mr Grongitts was very bored. He decided he would not visit another barbecue party for at least ... weeks. 4 / 6

speculation3 Gina discovered a new band on the internet. It's called 'This will destroy you' and she immediately purchased their debut album. As soon as she can afford it she intends to buy ... more of their albums. 3 / 4

speculation4 Amtrak operates trains in the US. The passengers know that delays are common. Indeed, yesterday's 8:30 Amtrak train from NYC to Boston was ... minutes late. 27 / 37

Appendix D: Items experiment 2

beer John is a man from the the US. John lives in the same apartment building as Anton. About an hour ago, when asked about it, Anton said that / Anton out of the blue said that John drank ... beers last month. 15 / 25

birthday Samantha is a 14-year-old girl from the US. Samantha has an aunt, Caroline. On Wednesday, when asked about it, Caroline said that / Caroline out of the blue said that Samantha attended ... birthday parties last year. 5 / 8

books Nathalie is a woman from the US. Nathalie often has dinner with Alice. A few days ago, when asked about it, Alice said that / Alice out of the blue said that Nathalie read. ... books last year. 7 / 11

burger Joseph is a man from the US. Joseph has a girlfriend, Sue. Yesterday, when asked about it, Sue said that / Sue out of the blue said that Joseph ate ... burgers last month. 6 / 9

calls Lisa is a woman from the US. Lisa has a stepmother, Mona. Today, when asked about it, Mona said that / Mona out of the blue said that Lisa made ... phone calls last week. 17 / 26

candy Scott is a 12-year-old from the US. Scott has a classmate, Matt. Today, when asked about it, Matt said that / Matt out of the blue said that Scott ate ... candy bars last week. 9 / 14

car Jeff is a man from the US. Jeff lives across the street from Amy. A few minutes ago, when asked about it, Amy said that / Amy out of the blue said that Jeff spent ... hours in his car last week. 11 / 16

cards Wanda is a woman from the US. Wanda carpools with Adam. Last Saturday, when asked about it, Adam said that / Adam out of the blue said that Wanda has ... cards in her wallet. 6 / 8

chairs Rob and Wendy are a couple from the US. Rob and Wendy have a friend, Katie. The other day, Katie, when asked about it, said that / Katie out of the blue said that Rob and Wendy own ... chairs. 8 / 12

class Erin is a first grade student in primary school. Erin has an uncle, Josh. This morning, when asked about it, Josh said that / Josh out of the blue said that there are ... children in Erin's class. 23 / 44

coffee Andy is a man from the US. Andy has an aunt, Katherine. This afternoon, when asked about it, Katherine said that / Katherine out of the blue said that Andy drinks ... cups of coffee per day. 2 / 3

colleagues Linda is a woman from the US. Linda plays tennis with Beth. About a week ago, when asked about it, Beth said that / Beth out of the blue said that Linda has ... colleagues. 13 / 19

concert Zach is a man from the US. Zach has a brother, Jim. A few days ago, when asked about it, Jim said that / Jim out of the blue said that Zach went to . . . concerts last year. 3 / 5

cook Tony is a man from the US. Nick has a sister, Emily. This afternoon, when asked about it, Emily said that / Emily out of the blue said that Tony cooked . . . meals at home last month. 36 / 101

cousin Jess is a woman from the US. Jess takes sewing classes with Anna. On Tuesday, when asked about it, Anna said that / Anna out of the blue said that Jess has . . . cousins. 6 / 9

date Trey and Tina are a couple from the US. Trey and Tina live next door to Paul. The other day, when asked about it, Paul said that / Paul out of the blue said that Trey and Tina hired a babysitter . . . times last year. 24 / 52

dishwasher Lily is a woman from the US. Lily has a nephew, Bob. On Monday, when asked about it, Bob said that / Bob out of the blue said that Lily ran her dishwasher . . . times last month. 18 / 27

dog Hugh is a man from the US. Hugh has a neighbor, Jenn. Just now, when asked about it, Jenn said that / Jenn out of the blue said that Hugh walked his dog . . . times last week. 9 / 13

football Kyle is a teenager from the US. Kyle has a friend, Wade. A few minutes ago, when asked about it, Wade said that / Wade out of the blue said that Kyle had football practice . . . times last month. 10 / 15

friends Lelia is a woman from the US. Lelia lives around the corner from Brad. Tonight, when asked about it, Brandon said that / Brandon out of the blue said that Lelia has . . . friends. 9 / 13

hair Betty is a woman from the US. Betty works at an office with David. Tonight, when asked about it, David said that / David out of the blue said that Betty washed her hair . . . times last month. 18 / 29

keys Brendan is a man from the US. Brendan has a gym buddy, Ryan. Just now, Ryan when asked about it, Ryan said that / out of the blue said that Brendan has . . . keys on his keychain. 5 / 7

laundry Peter is a man from the US. Peter shares an apartment with Jeffrey. Two days ago, when asked about it, Jeffrey said that / Jeffrey out of the blue said that Peter washed . . . loads of laundry last month. 7 / 11

movie Nick is a man from the US. Nick went to school with Stephanie. Yesterday, when asked about it, Stephanie said that / Stephanie out of the blue said that Nick saw . . . movies last year. 19 / 31

orderonline Robert is a man from the US. Robert has a co-worker, Margaret. About a week ago, when asked about it, Margaret said that / Margaret out of the blue said that Robert ordered something online . . . times last year. 24 / 78

phone Jill is a woman from the US. Jill has a best friend, Kevin. A few hours ago, when asked about it, Kevin said that / Kevin out of the blue said that Jill spent . . . hours on her phone last week. 15 / 24

plane Gary is a man from the US. Gary has a cousin, Alexander. On Tuesday, when asked about it, Alexander said that / Alexander out of the blue said that Gary was on . . . flights last year. 2 / 4

plants Pauline is a woman from the US. Pauline is in a reading group with Jack. Last Saturday, when asked about it, Jack said that / Jack out of the blue said that Pauline has . . . house plants. 4 / 7

restaurants Sarah is a woman from the US. Sarah has an acquaintance, Eric. Last week, when asked about it, Eric said that / Eric out of the blue said that Sarah went to eat out in a restaurant . . . times last year. 24 / 49

shoes Melanie is a woman from the US. Melanie has a colleague, Amber. This morning, when asked about it, Amber said that / Amber out of the blue said that Melanie owns . . . pairs of shoes. 12 / 18

takeout Ralph is a man from the US. Ralph has an uncle, Harry. A few hours ago, when asked about it, Harry said that / Harry out of the blue said that Ralph ordered take-out . . . times last month. 7 / 11

tshirts Liam is a man from the US. Liam lives down the street from Rebecca. Last week, when asked about it, Rebecca said that / Rebecca out of the blue said that Liam has . . . T-shirts. 13 / 21

vacuum Patricia is a woman from the US. Patricia lives next-door to Nora. On Monday, when asked about it, Nora said that / Nora out of the blue said that Patricia vacuumed . . . times last month. 6 / 9

wine Gilly is a woman from the US. Gilly has a roommate, Denise. About an hour ago, when asked about it, Denise said that / Denise out of the blue said that Gilly drank . . . glasses of wine last month. 8 / 13

workout Jennifer is a woman from the US. Jennifer has a sister, Holly. On Wednesday, when asked about it, Holly said / Holly out of the blue said that Jennifer worked out . . . times last month. 11 / 16

Appendix E: Items experiment 3

beer John is a man from the the US. John lives in the same apartment building as Anton. About an hour ago at the beach, Anton said to me / stood up and said to everyone that John drank . . . beers last month. 15 / 25

birthday Samantha is a 14-year-old girl from the US. Samantha has an aunt, Caroline. On Wednesday at the library, Caroline said to me / stood up and said to everyone that Samantha attended . . . birthday parties last year. 5 / 8

books Nathalie is a woman from the US. Nathalie often has dinner with Alice. A few days ago at the bus, Alice said to me / stood up and said to everyone that Nathalie read . . . books last year. 7 / 11

burgers Joseph is a man from the US. Joseph has a girlfriend, Sue. Yesterday at the local bar, Sue said to me / stood up and said to everyone that Joseph ate . . . burgers last month. 6 / 9

calls Lisa is a woman from the US. Lisa has a stepmother, Mona. Today at the post office, Mona said to me / stood up and said to everyone that Lisa made . . . phone calls last week. 17 / 26

candy Scott is a 12-year-old from the US. Scott has a classmate, Matt. Today at the playground, Matt said to me / stood up and said to everyone that Scott ate . . . candy bars last week. 9 / 14

car Jeff is a man from the US. Jeff lives across the street from Amy. A few minutes ago at a work dinner, Amy said to me / stood up and said to everyone that Jeff spent . . . hours in his car last week. 11 / 16

cards Wanda is a woman from the US. Wanda carpools with Adam. Last Saturday at our drama group, Adam said to me / stood up and said to everyone that Wanda has . . . cards in her wallet. 6 / 8

chairs Rob and Wendy are a couple from the US. Rob and Wendy have a friend, Katie. The other day at the shop, Katie said to me / stood up and said to everyone that Rob and Wendy own . . . chairs. 8 / 12

class Erin is a first grade student in primary school. Erin has an uncle, Josh. This morning at the office, Josh said to me / stood up and said to everyone that there are . . . children in Erin's class. 23 / 44

coffee Andy is a man from the US. Andy has an aunt, Katherine. This afternoon at the museum, Katherine said to me / stood up and said to everyone that Andy drinks . . . cups of coffee per day. 2 / 3

colleagues . Linda is a woman from the US. Linda plays tennis with Beth. About a week ago at the mall, Beth said to me / stood up and said to everyone that Linda has . . . colleagues 13 / 19

concert	Zach is a man from the US. Zach has a brother, Jim. A few days ago at work, Jim said to me / stood up and said to everyone that Zach went to . . . concerts last year. 3 / 5
cook	Tony is a man from the US. Nick has a sister, Emily. This afternoon at our pottery class, Emily said to me / stood up and said to everyone that Tony cooked . . . meals at home last month. 36 / 101
cousin	Jess is a woman from the US. Jess takes sewing classes with Anna. On Tuesday at the market, Anna said to me / stood up and said to everyone that Jess has . . . cousins. 6 / 9
dates	Trey and Tina are a couple from the US. Trey and Tina live next door to Paul. The other day at the swimming pool, Paul said to me / stood up and said to everyone that Trey and Tina hired a babysitter . . . times last year. 24 / 52
dishwasher	Lily is a woman from the US. Lily has a nephew, Bob. On Monday at the train, Bob said to me / stood up and said to everyone that Lily ran her dishwasher . . . times last month. 18 / 27
dog	Hugh is a man from the US. Hugh has a neighbor, Jenn. Just now at the store, Jenn said to me / stood up and said to everyone that Hugh walked his dog . . . times last week. 9 / 13
football	Kyle is a teenager from the US. Kyle has a friend, Wade. A few minutes ago at school, Wade said to me / stood up and said to everyone that Kyle had football practice . . . times last month. 10 / 15
friends	Lelia is a woman from the US. Lelia lives around the corner from Brad. Tonight at our family dinner, Brandon said to me / stood up and said to everyone that Lelia has . . . friends. 9 / 13
hair	Betty is a woman from the US. Betty works at an office with David. Tonight at the restaurant, David said to me / stood up and said to everyone that Betty washed her hair . . . times last month. 18 / 29
keys	Brendan is a man from the US. Brendan has a gym buddy, Ryan. Just now on the street, Ryan said to me / stood up and said to everyone that Brendan has . . . keys on his keychain. 5 / 7
laundry	Peter is a man from the US. Peter shares an apartment with Jeffrey. Two days ago at our school reunion, Jeffrey said to me / stood up and said to everyone that Peter washed . . . loads of laundry last month. 7 / 11
movies	Nick is a man from the US. Nick went to school with Stephanie. Yesterday at my party, Stephanie said to me / stood up and said to everyone that Nick saw . . . movies last year. 19 / 31

phone Jill is a woman from the US. Jill has a best friend, Kevin. A few hours ago at our work meeting, Kevin said to me / stood up and said to everyone that Jill spent . . . hours on her phone last week. 15 / 24

plane Gary is a man from the US. Gary has a cousin, Alexander. On Tuesday at the baseball game, Alexander said to me / stood up and said to everyone that Gary was on . . . flights last year. 2 / 4

orderonline Robert is a man from the US. Robert has a co-worker, Margaret. About a week ago at the cafeteria, Margaret said to me / stood up and said to everyone that Robert ordered something online . . . times last year. 24 / 78

plants Pauline is a woman from the US. Pauline is in a reading group with Jack. Last Saturday the park, Jack said to me / stood up and said to everyone that Pauline has . . . house plants. 4 / 7

restaurants Sarah is a woman from the US. Sarah has an acquaintance, Eric. Last week at our choir rehearsal, Eric said to me / stood up and said to everyone that Sarah went to eat out in a restaurant . . . times last year. 24 / 49

shoes Melanie is a woman from the US. Melanie has a colleague, Amber. This morning, at the subway, Amber said to me / stood up and said to everyone that Melanie owns . . . pairs of shoes. 12 / 18

takeout Ralph is a man from the US. Ralph has an uncle, Harry. A few hours ago at the fair, Harry said to me / stood up and said to everyone that Ralph ordered take-out . . . times last month. 7 / 11

tshirts Liam is a man from the US. Liam lives down the street from Rebecca. Last week at the conference, Rebecca said to me / stood up and said to everyone that Liam has . . . T-shirts. 13 / 21

vacuum Patricia is a woman from the US. Patricia lives next-door to Nora. On Monday at the square, Nora said to me / stood up and said to everyone that Patricia vacuumed . . . times last month. 6 / 9

wine Gilly is a woman from the US. Gilly has a roommate, Denise. About an hour ago at the movies, Denise said to me / stood up and said to everyone that Gilly drank . . . glasses of wine last month. 8 / 13

workout Jennifer is a woman from the US. Jennifer has a sister, Holly. On Wednesday at the cafe, Holly said to me / stood up and said to everyone that Jennifer worked out . . . times last month. 11 / 16

Appendix F: Experiments 2 and 3 pre-test

The pre-test for Experiments 2 and 3 followed that of Experiment 1 and was used to estimate participants' priors. The pre-test participants (N=20 after elimination of participants who failed the catch trials) did not participate in any other experiment in this paper. They were recruited on Amazon Mechanical Turk and paid $4. Each participant saw the same 35 target scenarios from the Experiment 2&3 materials. They were asked in a free-response task to answer questions about things like the number of objects in someone's possession or the frequency of events in someone's life (*Jeff is a man from the US. How many hours did Jeff spend in his car last week?*). Participants were asked to provide their 'best guess' as well as a maximum and minimum. The distribution of the 20 responses for 'best guess' for each item is shown in Appendix Figures 2-3.

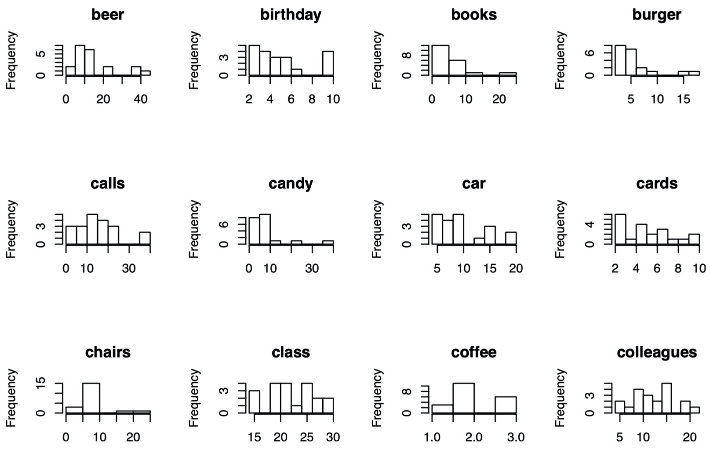

Figure 2: Frequencies of different values in pre-test norming for Experiments 2 and 3, *beer-colleagues*

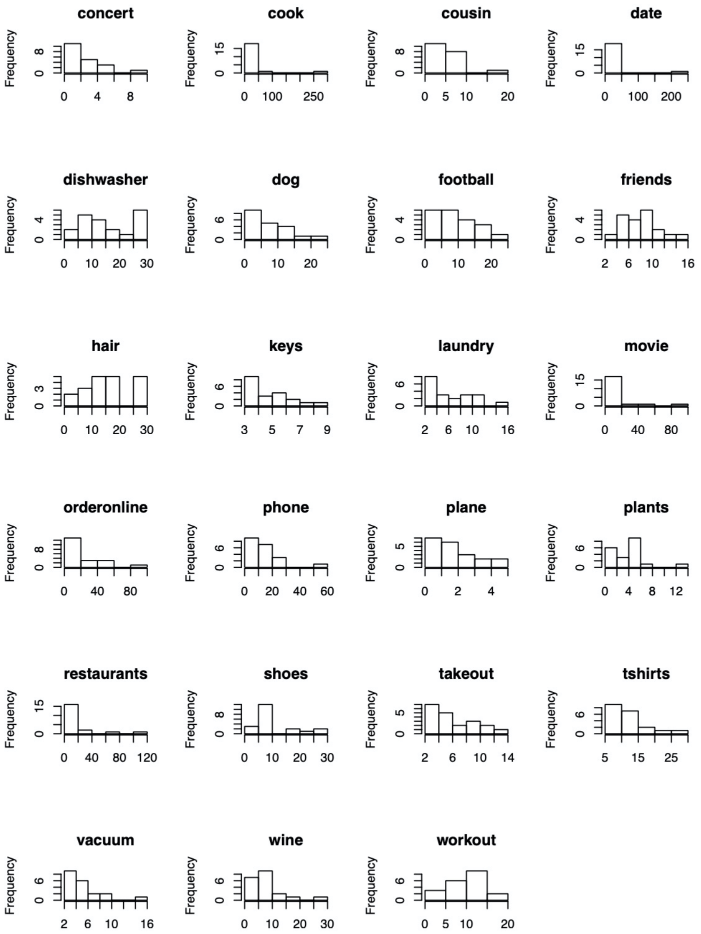

Figure 3: Frequencies of different values in pre-test norming for Experiment 2 and 3, *concert-workout*

Appendix G: Items Experiment 4

Conditions below correspond to HIGH/MID/LOW.

1. Mary is a woman from the US. Mary lives next door to Paul. The other day at the swimming pool, Paul stood up and said to everyone / The other day, Paul announced / Paul thinks that Mary owns ... purses and handbags. 8 / 14

2. John is a man from the US. John lives in the same apartment building as Anton. About an hour ago at the beach, Anton stood up and announced to the crowd / About an hour ago, Anton out of the blue said to me / About an hour ago, when asked about it, Anton said that John drank ... beers last month. 22 / 39

3. Charlotte is a woman from the US. Charlotte has a sister, Emily. This afternoon at our pottery class, Emily stood up and said to everyone / This afternoon, Emily announced / Emily thinks that Charlotte has ... books at home. 24 / 49

4. Joseph is a man from the US. Joseph has a girlfriend, Sue. Yesterday at the local bar, Sue stood up and announced to the crowd / Yesterday Sue announced / Yesterday, when asked about it, Sue said that Joseph ate ... burgers last month. 8 / 13

5. Scott is a 12-year-old boy from the US. Scott has a classmate, Matt. Today at the playground, Matt stood up and said to everyone / Today Matt announced / Matt thinks that Scott ate ... candy bars last month. 9 / 14

6. Wanda is a woman from the US. Wanda carpools with Adam. Last Saturday at our drama group, Adam stood up and announced to the crowd / Last Saturday, Adam out of the blue said to me / Last Saturday, when asked about it, Adam said that Wanda has ... cards in her wallet. 4 / 6

7. Rose is a 60-year-old woman from the US. Rose lives around the corner from Brad. Tonight at our family dinner, Brandon stood up and said to everyone / Tonight Brandon announced / Tonight, when asked about it, Brandon said that Rose has owned ... cars. 4 / 7

8. Rob and Wendy are a couple from the US. Rob and Wendy have a friend, Katie. The other day at the shop, Katie stood up and announced to the crowd / The other day, Katie out of the blue said to me / Katie thinks that Rob and Wendy own ... chairs. 7 / 11

9. Erin is a first grade student in primary school. Erin has an uncle, Josh. This morning at the office, Josh stood up and said to everyone / This morning, Josh announced / This morning, when asked about it, Josh said that there are ... children in Erin's class. 22 / 26

10. Andy is a man from the US. Andy has an aunt, Katherine. This afternoon at the museum, Katherine stood up and announced to the crowd / This afternoon, Katherine announced / Katherine thinks that Andy drinks ... cups of coffee per day. 3 / 4

11. Gary is a man from the US. Gary often has dinner with Alice. A few days ago at the bus, Alice stood up and said to everyone / A few days ago, Alice announced / A few days ago, when asked about it, Alice said that Gary owns ... coffee cups. 6 / 9

12. Linda is a woman from the US. Linda plays tennis with Beth. About a week ago at the mall, Beth stood up and announced to the crowd / About a week ago, Beth out of the blue said to me / About a week ago, when asked about it, Beth said that Linda has ... colleagues. 13 / 23

13. Jess is a woman from the US. Jess takes sewing classes with Anna. On Tuesday at the market, Anna stood up and said to everyone / On Tuesday, Anna announced / Anna thinks that Jess has ... cousins. 6 / 11

14. Sylvie is a woman from the US. Sylvie has an acquaintance, Eric. Last week at our choir rehearsal, Eric stood up and announced to the crowd / Last week, Eric out of the blue said to me / Last week, when asked about it, Eric said that Sylvie visits a doctor ... times a year. 3 / 6

15. Hugh is a man from the US. Hugh has a neighbor, Jenn. Just now at the store, Jenn stood up and said to everyone / Just now Jenn announced / Jenn thinks that Hugh walked his dog ... times last week. 7 / 9

16. Rebecca is a woman from the US. Rebecca has a stepmother, Mona. Today at the post office, Mona stood up and announced to the crowd / Today Mona announced / Today, when asked about it, Mona said that Rebecca owns ... pairs of earrings. 13 / 23

17. Sidney is a man from the US. Sidney has a brother, Jim. A few days ago at work, Jim stood up and said to everyone / A few days ago, Jim announced / A few days ago, when asked about it, Jim said that Sidney receives ... emails each day. 17 / 32

18. George is a single 30-year-old man from the US. George has a cousin, Gregory. On Tuesday at the baseball game, Gregory stood up and announced to the crowd / On Tuesday, Gregory out of the blue said to me / Gregory thinks that George has been on ... first dates in his life. 15 / 32

19. Noah and Ava are an 80-year-old couple from the US. Noah and Ava live across the street from Amy. A few minutes ago at a work dinner, Amy stood up and said to everyone / A few minutes ago, Amy announced / A few minutes ago, when asked about it, Amy said that Noah and Ava have ... grandchildren. 8 / 13

20. Betty is a woman from the US. Betty works at an office with David. Tonight at the restaurant, David stood up and announced to the crowd / Tonight David out of the blue said to me / David thinks that Betty washed her hair ... times last month. 16 / 24

21. Brendan is a man from the US. Brendan has a gym buddy, Ryan. Just now on the street, Ryan stood up and said to everyone / Just now Ryan out of the blue said to me / Just now, Ryan when asked about it, Ryan said that Brendan has ... keys on his key chain. 6 / 9

22. Henry is a man from the US. Henry has an aunt, Caroline. On Wednesday at the library, Caroline stood up and announced to the crowd / On Wednesday, Caroline announced / Caroline thinks that Henry owns ... lamps. 4 / 6

23. Peter is a man from the US. Peter shares an apartment with Jeffrey. Two days ago at our school reunion, Jeffrey stood up and said to everyone / Two days ago, Jeffrey out of the blue said to me / Jeffrey thinks that Peter washed ... loads of laundry last month. 6 / 9

24. Caroline is an employee at a US company. Caroline has a sister, Holly. On Wednesday at the cafe, Holly stood up and announced to the crowd / On Wednesday, Holly out of the blue said to me / On Wednesday, when asked about it, Holly said that Caroline had ... meetings last week. 4 / 6

25. Nick is a man from the US. Nick went to school with Stephanie. Yesterday at my party, Stephanie stood up and said to everyone / Yesterday Stephanie announced / Stephanie thinks that Nick saw ... movies last year. 23 / 45

26. Robert is a man from the US. Robert has a co-worker, Margaret. About a week ago at the cafeteria, Margaret stood up and announced to the crowd / About a week ago, Margaret out of the blue said to me that / About a week ago, when asked about it, Margaret said that Robert ordered something online ... times last year. 29 / 63

27. Jasper is a 70-year-old man from the US. Jasper has a nephew, Bob. On Monday at the train, Bob stood up and said to everyone / On Monday, Bob out of the blue said to me / On Monday, when asked about it, Bob said that Jasper has owned ... pets in his life. 7 / 12

28. Paul and Jacky are a couple from the US. Paul and Jacky live across the hall from Steve. Last week at the barbecue, Steve stood up and announced to the crowd / Last week, Steve announced / Steve thinks that Paul and Jacky own ... throw pillows. 6 / 9

29. Pauline is a woman from the US. Pauline is in a reading group with Jack. Last Saturday the park, Jack stood up and said to everyone / Last Saturday, Jack out of the blue said to me / Pauline is in a reading group with Jack. Jack thinks that Pauline has ... house plants. 6 / 11

30. Ann is a woman from the US. Ann has a house cleaner Maya. Last week at the yard sale, Maya stood up and announced to the crowd / Last week, Maya out of the blue said to me / Maya thinks that Ann eats ... sandwiches in a week. 4 / 7

31. Melanie is a woman from the US. Melanie has a colleague, Amber. This morning at the subway, Amber stood up and said to everyone / This morning, Amber announced / This morning, when asked about it, Amber said that Melanie owns ... pairs of shoes. 16 / 28

32. Olivia is a 20-year-old woman from the US. Olivia has a housemate, Michael. Yesterday evening at the pub, Michael stood up and announced to the crowd / Yesterday evening, Michael out of the blue said to me / Yesterday evening, when asked about it, Michael said that Olivia has had ... smartphones in her life. 4 / 6

33. Ryan is a 50-year-old man from the US. Ryan has a golf buddy, Chris. This morning at the clubhouse, Chris stood up and said to everyone / This morning, Chris out of the blue said to me / Chris thinks that Ryan has received a speeding ticket ... times in his life. 7 / 16

34. Samantha is a woman from the US. Samantha works in the same company as Ronny. On Saturday at the company picnic, Ronny stood up and announced to the crowd / On Saturday, Ronny announced / On Saturday, when asked about it, Ronny said that Samantha has visited ... states. 8 / 14

35. James is a 5-year-old child from the US. James has a friend, Wade. A few minutes ago at school, Wade stood up and said to everyone / A few minutes ago, Wade out of the blue said to me / Wade thinks that James has ... stuffed animals. 9 / 17

36. Ralph is a man from the US. Ralph has an uncle, Harry. A few hours ago at the fair, Harry stood up and announced to the crowd / A few hours ago, Harry out of the blue said to me / Harry thinks that Ralph ordered take-out ... times last month. 11 / 18

37. Liam is a man from the US. Liam lives down the street from Rebecca. Last week at the conference, Rebecca stood up and said to everyone / Last week, Rebecca announced / Last week, when asked about it, Rebecca said that Liam has ... T-shirts. 18 / 28

38. Patricia is a woman from the US. Patricia lives next-door to Nora. On Monday at the square, Nora stood up and announced to the crowd / On Monday, Nora out of the blue said to me / Nora thinks that Patricia vacuumed ... times last month. 6 / 11

39. Zach is a man from the US. Zach has a best friend, Kevin. A few hours ago at our work meeting, Kevin stood up and said to everyone / A few

hours ago, Kevin out of the blue said to me / A few hours ago, when asked about it, Kevin said that Zach played ... hours of video games last week. 13 / 22

40. Alexander is a 1-year-old baby from the US. Alexander has a godmother Liane. This weekend at the gymboree, Liane stood up and announced to the crowd / This weekend, Liane announced / Liane thinks that Alexander wakes up ... times a night. 3 / 4

41. Roy and Emma are a couple from the US. Roy and Emma are in a book club with Kenny. A few weeks ago at the pub, Kenny stood up and said to everyone / A few weeks ago, Kenny announced / A few weeks ago, when asked about it, Kenny said that Roy and Emma have ... windows in their 2-bedroom apartment. 4 / 8

42. Gilly is a woman from the US. Gilly has a roommate, Denise. About an hour ago at the movies, Denise stood up and announced to the crowd / About an hour ago, Denise out of the blue said to me / Denise thinks that Gilly drank ... glasses of wine last month. 16 / 31

Appendix H: Experiment 4 pre-test

We conducted a pre-test of 60 candidate items (*Liam is a man from the US. How many T-shirts do you think Liam has?*). Even though some items were adapted from Experiments 2-3, we wanted to establish up-to-date low/high values, given that estimates of typical behavior might have changed over the course of the COVID-19 pandemic. Participants (n=24) after elimination of participants who failed the catch trials) were recruited from Prolific and paid $3 for their time. None participated in any other experiment in this paper. Participants were asked to provide their 'best guess' as well as a maximum and minimum. The goal was to find a set of items whose mean of the maximum was less than 1 standard deviation from the mean 'best guess' in order to ensure that the value we selected as the 'high' response was still a plausible value. That criterion eliminated 17 items, and we also eliminated a further outlier item whose mean maximum was proportionally much larger than the mean best guess compare to other items. This left 42 items for the experiment.

Appendix I: Experiment 4 fillers

For this experiment, we used 12 fillers with correct answers to serve as attention checks.

1. Ben is a man from the US. Ben has a stepfather Daniel. Select the higher number. Daniel thinks that Ben has ... power outlets in his office. 5 / 9
2. Dexter is a man from the US. Dexter has an aunt Emily. Select the lower number. The other day, Emily announced that Dexter shaves ... times each month. 14 / 25
3. Tony is a man from the US. Select the higher number. Tony has an uncle Max. This morning at the bakery, Max stood up and said to everyone that Tony cooked ... meals at home last month. 19 / 39
4. Ted is a manager at a US company. Select the lower number. Ted has a cousin Kyle. Yesterday, when asked about, Kyle said that Ted held ... job interviews last year. 17 / 39
5. Lily is a woman from the US. Lily has a sister Tina. Choose the odd number. About an hour ago, Tina out of the blue said to me that Lily ran her dishwasher ... times last month. 19 / 30
6. Lelia is a woman from the US. Leila has a brother Andrew. Choose the odd number. About an hour ago at the bookstore, Andrew announced that Lelia has ... friends. 12 / 27
7. Nathalie is a woman from the US. Choose the even number. Nathalie has a hairdresser Lisa. Choose the even number. Lisa thinks that Nathalie read ... books last year. 11 / 30
8. Sarah is a woman from the US. Choose the even number. Sarah has an aromatherapist Jill. Last weekend, Jill announced that Sarah had ... exams as a senior in college. 24 / 33
9. Sophia is a woman from the US. Sophia has a dogwalker Roger. Pick the bigger value. Yesterday at the lake, Roger stood up and said to everyone that Sophia mowed her lawn ... times in the last year. 20 / 42
10. Jennifer is a woman from the US. Pick the smaller value. Jennifer has a psychiatrist Elizabeth. Last week, when asked about it, Elizabeth said that Jennifer worked out ... times last month. 17 / 40
